# *Wolf-hound* vs. *sled-dog*: neurolinguistic evidence for semantic decomposition in the recognition of German noun-noun compounds

**DOI:** 10.3389/fpsyg.2023.1173352

**Published:** 2023-08-17

**Authors:** Anna Czypionka, Mariya Kharaman, Carsten Eulitz

**Affiliations:** Department of Linguistics, University of Konstanz, Konstanz, Germany

**Keywords:** animacy, compound, N400, word recognition, semantic decomposition

## Abstract

Animacy is an intrinsic semantic property of words referring to living things. A long line of evidence shows that words with animate referents require lower processing costs during word recognition than words with inanimate referents, leading among others to a decreased N400 amplitude in reaction to animate relative to inanimate objects. In the current study, we use this animacy effect to provide evidence for access to the semantic properties of constituents in German noun-noun compounds. While morphological decomposition of noun-noun compounds is well-researched and illustrated by the robust influence of lexical constituent properties like constituent length and frequency, findings for semantic decomposition are less clear in the current literature. By manipulating the animacy of compound modifiers and heads, we are able to manipulate the relative ease of lexical access strictly due to intrinsic semantic properties of the constituents. Our results show additive effects of constituent animacy, with a higher number of animate constituents leading to gradually attenuated N400 amplitudes. We discuss the implications of our findings for current models of complex word recognition, as well as stimulus construction practices in psycho-and neurolinguistic research.

## 1. Introduction

Animacy describes the property of certain things that we perceive as “having a soul”^1^ or more simply put as being alive; the least ambiguous examples involving (vertebrate) animals including fellow humans. The distinction between animate and inanimate entities shapes many different areas of cognition.

With respect to language, animacy belongs to the semantic properties making up the meaning of a word, and is arguably one of the most striking and influential of its semantic properties. In natural language, words referring to animate entities are highly salient compared to words referring to inanimate entities. This is visible in the special “treatment” that animate entities get in the world's languages. In sentence production, speakers go out of their way to produce animates in early sentence positions and make them the subjects, rather than the objects, of sentences, often at the cost of syntactic simplicity. In sentence comprehension, the animacy of the arguments is one of the crosslinguistically most robust cues for understanding who did what to whom. Even at the single-word level, words referring to animate entities are recognized more quickly than words referring to inanimate entities, thanks to their high saliency.

In this paper, we present an EEG study using this processing benefit of animates over inanimates to answer a long-standing question in single-word recognition research. Our question concerns the recognition of compound words consisting of two nouns, and whether the semantic properties of both constituent nouns are routinely accessed during compound recognition.

In the following, we provide an overview of the role of animacy in language processing, followed by a literature overview of the processing of noun-noun compounds. We will then give an outline of how we use animacy to monitor semantic decomposition in compound recognition, and formulate our research questions and hypotheses in more detail before presenting the results of our study.

## 2. Background

### 2.1. Animacy

The semantic property of animacy is a strong influence in many languages of the world, both at the single-word and sentence level. Animates and inanimates are referred to with different interrogative pronouns (*who* and *what*, respectively, in English), and differ in number marking (Croft, 1990; Corbett, 2000; Haspelmath, 2013). In Differential Object Marking (DOM) languages (like Hindi or Spanish), overt object case marking is only obligatory for a particular semantic class of nouns, with animacy or even humanness being a frequent classification (Bossong, 1985, 1991; Næss, 2004; Malchukov, 2008). Furthermore, the morphological makeup of nouns via case syncretism is shaped by animacy in a complex interaction with agentivity, biological and grammatical gender (Krifka, 2009). A thorough overview of crosslinguistic animacy effects can be found in Yamamoto (1999).

The special status of the animate-inanimate distinction in human language is mirrored in language processing.^2^ During language acquisition, the distinction between animates and inanimates develops early in life (Opfer and Gelman, 2010), an observation that also holds for autistic children (Rutherford et al., 2006). In the case of language deficits, different studies report category-specific deficits affecting only one semantic subclass, while other semantic subclasses are spared. Animacy is one of the relevant semantic subclasses; for example, a patient may exhibit impaired naming for animals, but not for fruit and vegetables (Caramazza and Shelton, 1998) or artifacts (see Capitani et al., 2003; Caramazza and Mahon, 2003 for overviews).

In adult language processing, there is also a host of evidence for a distinction between animates and inanimates. In general, findings suggest that lexical access is less costly for animates than for inanimates. Behavioral studies in multiple languages show that reaction times are shorter for animates than inanimates in word and picture naming (Janyan and Andonova, 2011), semantic categorization and lexical decision tasks (Bonin et al., 2019), and for ink color naming in a Stroop task adaptation (Bugaiska et al., 2019). In addition, animates are remembered better than inanimates, both in free recall and paired-associate tasks (Nairne et al., 2013; VanArsdall et al., 2015); these findings are unlikely to be reduced to categorical recall strategies (VanArsdall et al., 2017). Further support comes from Bonin et al. (2014), who found that animates are remembered better than inanimates, both for word and picture stimuli, and word recall and recognition tasks (see also Bonin et al., 2015 for replication and added detail). Neurolinguistic evidence^3^ for the animate-inanimate distinction includes differential BOLD responses for animals relative to manipulable objects (Anzellotti et al., 2011) and differences in the EEG spectral power (Verkhlyutov et al., 2014). In EEG studies, Sitnikova et al. (2006) found an increased anterior negativity for animals relative to tools, and left-posterior negativity for tools relative to animals, between 200 and 600 ms. They interpret their findings as evidence for feature-based organization of semantic knowledge. Proverbio et al. (2007) investigated images of animate and inanimate stimuli in a non-verbal categorization task. Stimulus pairs were presented and participants had to judge if they belonged to the same or to different semantic categories (animals or artifacts). Compared to artifacts, animates showed shorter reaction times, higher accuracy, a larger P300 amplitude and a reduced N400 amplitude. The authors conclude that in contrast to animates, manipulable objects lead to the activation of areas associated with motor representation (see, however, findings by Ković et al., 2009, suggesting no N400 amplitude differences for animates relative to inanimates.).

In the processing of sentences, the animacy of arguments is the central semantic cue for argument role assignment (e.g., MacDonald et al., 1994; Trueswell et al., 1994; Weckerly and Kutas, 1999; Frisch and Schlesewsky, 2001; Kuperberg, 2007; Branigan et al., 2008; Bornkessel-Schlesewsky et al., 2011; Paczynski and Kuperberg, 2011; Czypionka, 2014), interacting with the processing of number agreement (Bamyacı et al., 2014) and case marking (Verhoeven, 2014; Czypionka and Eulitz, 2018). The prominent role of argument animacy in sentence processing is reflected in its central role in models of sentence processing, where it is associated with the assignment of thematic roles (see, among others, Levelt, 1993; Bornkessel-Schlesewsky and Schlesewsky, 2006, 2009, 2013; Hagoort, 2007, 2016; Kuperberg, 2007 for different approaches to sentence comprehension and production).^4^

In sum, animacy is an intrinsic semantic property of a word's referent that influences all levels of language processing. Words with animate referents are highly salient in the sentence and discourse context. Lexical access is less costly for words with animate referents than for words with inanimate referents. This is reflected in shorter reaction times and reduced N400 amplitudes for animates relative to inanimates. This makes animacy a useful tool for investigating the role of semantics in single-word processing, all the more as it is an intrinsic property that does not depend on context.^5^ In the following, we will outline how these properties can be informative for questions related to compound processing, in particular with respect to semantic decomposition.

### 2.2. Compounds

Compounds are words consisting of more than one constituent; in the context of this paper, we will refer exclusively to noun-noun compounds unless specifically mentioned otherwise.^6^ These are words like *gunpowder* or *garden hose*. In English, these words appear both as a single orthographic unit (*gunpowder*) and as two adjacent nouns (*garden hose*), with little semantic difference between both options. In other languages like German, however, orthographic rules for noun-noun compounds demand that they appear as one orthographic unit (*Schieß.pulver* “gunpowder”, *Garten.schlauch* “garden hose”, dots marking the constituent boundaries are not part of the German orthography and are only inserted here for clarity).

The lexical category, syntactic features, and main semantic properties of the compound depend on the lexical head, which is always the last (in our case, second) constituent in German compounds (as it is in most English compounds): *Schlittenhund*, “sled dog” is a kind of dog, not of sled, whereas *Hundeschlitten*, “dog sled”, is a kind of sled, not a kind of dog. The first constituent is the modifier, extending and modifying the meaning of the lexical head: *Schlittenhund* is the specific kind of dog that pulls sleds, whereas *Hundeschlitten* is the specific kind of sled that is pulled by dogs.

Compounds can be semantically transparent or opaque. With semantically transparent compounds like *Pferdedecke* “horse rug”, the full-form meaning can easily be inferred from combining the meanings of its constituents in a straightforward manner—a horse rug is a rug or blanket used to cover a horse. With semantically opaque compounds, the full-form meaning cannot be inferred by simply combining the constituent meanings (*Windbeutel*, literally “wind bag”, is not a bag full of wind, but rather a cream-puff-like pastry).

In psycho- and neurolinguistic research, compounds are mostly studied with the focus on the nature of their lexical entries and lexical access. The main overarching research question in this literature is the amount and nature of compound decomposition, i.e., whether compounds are stored and accessed via their full-form meaning, or whether this meaning is calculated from the constituents when compounds are encountered. Related questions are concerned with whether decomposition occurs in a similar manner for all kinds of compounds, and which kinds of constituent information is accessed during decomposition.^7^

According to full-listing models (Butterworth, 1983; Bybee, 1995), known words are always stored and accessed in their full form in the lexicon. In contrast, full-parsing models (Taft and Forster, 1975; Libben et al., 1999; McKinnon et al., 2003; Taft, 2004; Taft and Ardasinski, 2006; Taft and Nguyen-Hoan, 2010) propose morphological decomposition for all complex words. Another proposal is that decomposition and full-form access are both a part of complex word recognition, but happen at different points in time (e.g., according to the supralexical model by Giraudo and Grainger, 2001, where full-form access precedes decomposition). Finally, dual-route models allow for both full-form access and decomposition before access, (e.g., Augmented Addressed Morphology Model by Caramazza et al., 1988, or the Morphological Race Model by Schreuder and Baayen, 1995; Baayen and Schreuder, 1999); the multiple-route model by Kuperman et al. (2009) also allows for parallel access via multiple and interactive routes. Which route ultimately leads to identification depends on the words' familiarity, its semantic transparency, and the frequencies of its constituents and full-form, among other factors.

The number and variety of different accounts of lexical access already hints at the very different findings with respect to compound recognition in the literature.^8^ In general, a strong point in favor of decomposition is when properties of the modifier (in addition to full-form and head properties) can be shown to influence compound recognition.

While especially some earlier work argues against automatic decomposition of complex words (e.g., Sandra, 1990), the picture has become more nuanced over time, highlighting the important role of experimental paradigm, linguistic context and stimulus properties for eliciting compound decomposition. In an EEG study monitoring the processing of compounds in sentence reading, Stites et al. (2016) report enhanced late positivities for letter transpositions relative to non-transposed baselines. The effects of letter transpositions did not differ for transpositions within constituents and across constituent boundaries, suggesting that in this paradigm, full-form access offers the best explanation for the findings. Huang et al. (2020) report findings from a cross-modal priming study in Chinese. Primes were opaque compounds in a sentence context. Morphological priming was observed with neutral sentences, but not with sentences biasing toward the opaque meaning. These findings suggest that the extent to which compound constituents are accessed during sentence processing is influenced by the sentence context.

In contrast, many studies have shown at least some amount of decomposition for compounds during word recognition, often as a function of semantic transparency.

Libben et al. (2003) report repetition priming for both first and second constituents as speeding up compound recognition times, arguing for routine decomposition in both transparent and opaque compounds. A recent line of research has made use of reduction or enhancement of the mismatch negativity (MMN) amplitude in an oddball paradigm. MMN amplitude is reduced during combinatorial processing, but enhanced during full-form lexical access, making it a valuable tool for researching decomposition. For Chinese, Tsang et al. (2022) report a reduction of the MMN amplitude for transparent compounds relative to pseudocompounds, but equal MMN amplitudes for opaque and pseudocompounds. Zou et al. (2023) report an MMN amplitude reduction relative to the pseudoword baseline for low-frequency compounds, but not for high-frequency compounds. The authors of the respective studies explain their findings as showing that Chinese compounds are routinely decomposed. For transparent and for low-frequency compounds, combinatorial processing seems to be the dominant way of lexical access. For opaque and for high-frequency compounds, the MMN reduction from combinatorial processing is canceled out by the MMN enhancement due to full-form access, with both effects canceling each other out and leading to similar MMN amplitudes as in the processing of pseudowords.

One point supporting the idea of early decomposition is the fact that constituent frequency has an impact on compound recognition in a number of different languages [Juhasz et al. (2003), Andrews et al. (2004), Fiorentino and Poeppel (2007), Wang et al. (2010), and MacGregor and Shtyrov (2013) for English, Duñabeitia et al. (2007) for Basque and Spanish, Kuperman et al. (2009) for Dutch, Bronk et al. (2013) for German, and Hyönä and Pollatsek (1998) and Pollatsek et al. (2000) for Finnish]. The general direction of effects is a compound benefit, i.e., a processing advantage for compounds relative to simple words matched for full-form length and frequency. This strongly suggests that compounds are routinely decomposed during word recognition. It also (again) supports the idea that morphological decomposition is not *per se* costly, at least not so much as to override the processing benefit from more easily accessible (highly-frequent) constituents.

The above literature illustrating processing benefits for compounds depending on lexical properties of the constituents draws on data from a variety of languages and methods. Fiorentino and Poeppel (2007) found faster reaction times and different MEG signatures for compounds relative to simple words matched for length and frequency [see Crepaldi et al. (2013) and Fiorentino et al. (2014) for additional behavioral and EEG evidence in favor of compound decomposition]. MacGregor and Shtyrov (2013) manipulated frequency and transparency to investigate whether constituents are accessed during compound recognition, concluding that transparent compounds are accessed combinatorially with constituent and full-form properties both influencing lexical access, while high-frequency opaque compounds are accessed via their full form.

Some of the studies supporting morphological decomposition suggest a special role for the second constituent. Duñabeitia et al. (2007) monitored the processing of Basque compounds, manipulating compound headedness (unlike Germanic languages, but like, e.g., Italian, Basque allows both right- and left-headed compounds, allowing to disentangle effects of position from those of headedness). The authors found facilitation only for second, but not first constituents, interpreting their findings as showing routine decomposition which is however blind to semantics. In another study on Basque compounds, Vergara-Mart́ınez et al. (2009) used EEG measurements, manipulating the frequency of constituents and the compound headedness. They found that the N400 amplitude was larger for low- than high-frequency second constituents, while evidence for an influence of first constituent frequency was less clear.

Additional evidence for a privileged position of the second constituent in German compounds (where position is confounded with headedness) is provided by Holle et al. (2010), who report larger N400 amplitudes when heads (rather than modifiers) are exchanged for non-words.

Strong evidence in favor of lexical access to both heads and modifiers is presented by Bronk et al. (2013). In a series of lexical decision task experiments, they tested the recognition of German compounds against simple words matched for full-form length and frequency. Compounds came in two conditions, one with a highly frequent modifier, and the other with a low-frequency modifier. Results showed that compounds with high-frequency modifiers elicited shorter reaction times than compounds with low-frequency constituents or simple words. This finding was robust for both semantically transparent and opaque compounds; however, for opaque compound only, the constituent benefit was lost in the presence of difficult rather than easy pseudowords (i.e., with nonexisting combinations of two existing nouns). The authors describe this as evidence for early morphological decomposition, before access to the semantics of the full form, arguing against models assuming full-form access instead of or before decomposition. The findings also strongly support lexical access to modifiers.

In sum, there is ample evidence for morphological decomposition of noun-noun compounds, beginning early during word recognition. Lexical constituent properties like frequency and length influence processing cost, showing that the lexical entries of the constituents are accessed during compound recognition. However, the question remains whether semantic constituent properties are routinely accessed during compound recognition in a similar way to lexical constituent properties, and whether they have an influence on compound processing.

Compared to the vast literature on morphological decomposition of complex words, the literature on semantic decomposition is still smaller, and studies tend to focus on different aspects of semantic constituent properties. One approach is to focus on the influence of semantic transparency, comparing the processing of transparent vs. opaque complex words. Early priming studies (Sandra, 1990; Zwitserlood, 1994 for Dutch) report semantic priming of constituent meanings for transparent, but not for opaque compounds. While this suggests some amount of semantic access to constituents for transparent compounds, the findings were also interpreted as evidence against automatic full decomposition for all types of compounds, since opaque compounds seem to not be connected to their constituents at the semantic level (see also Pratarelli, 1995 for additional influences of length in English).

To assess the role of semantic transparency in derived words, Smolka et al. (2014) and Smolka and Eulitz (2018) used German complex verbs in a series of priming experiments. Verbs included both non-separable prefix verbs like *ver.stehen* (“to understand”) and separable particle verbs like *auf.stehen* (“to stand up”); like compounds, these complex verbs can be semantically transparent or opaque. They consistently found that priming from the verb stems was comparable for semantically opaque and transparent complex verbs, suggesting that the lexical representation of complex verbs is accessed via the verb base, irrespective of whether this verb base contributes to the full-form semantics of the complex verb. Koester et al. (2007) investigated the processing of acoustically presented German compounds using EEG. In their stimuli, the gender of the full form and the first constituent were either congruent or incongruent (in the German three-gendered system); this was manipulated for semantically transparent and opaque compounds. For incongruent gender only, they found an increase in the amplitude of the left anterior negativity (LAN), interpreted as evidence of morphological decomposition. Relative to opaque compounds, transparent compounds showed an increased negativity with a centroparietal maximum that occurred during the presentation of the head constituent. The authors interpreted their findings as showing semantic integration of constituents that had previously been accessed separately, arguing that transparent, but not opaque compounds need to be semantically integrated, which incurs additional processing costs. (These additional processing costs due to semantic integration for transparent compounds are not usually discussed in the literature reporting benefits for compounds relative to simple words; see above).

In a follow-up study, Koester et al. (2009) used German compounds consisting of three constituents, manipulating the plausibility of the second and third constituents. Implausible third constituents led to increased N400 amplitudes, as did implausible second constituents. The authors interpret these increased N400 amplitudes as showing the difficulty of lexical integration for implausible constituent combinations; furthermore, they argue that their findings show incremental lexical integration as morphologically complex words unfold. In a series of six lexical decision tasks, Ji et al. (2011) monitored the processing of English compounds that were semantically transparent (e.g., *rosebud*) or opaque (e.g., *hogwash*). In their experiments, they manipulated the likelihood of semantic decomposition (e.g., by adding easy or difficult to spot pseudowords, or by separating the two constituents by empty spaces or color markings). Like preceding studies, they found a compound processing advantage relative to length- and frequency-matched simple words. This advantage was initially visible for both transparent and opaque compounds, but held up only for transparent compounds when decomposition was encouraged. The authors interpret their findings as supporting semantic composition, with the opacity disadvantage showing a conflict between different potential meanings of opaque words.

In two behavioral experiments, Marelli and Luzzatti (2012) investigated the processing of Italian compounds, manipulating headedness, semantic transparency, and constituent frequency. Their results show that both constituent frequency influences recognition, and interacts with full-form properties and semantic transparency. The authors argue for an extension of multiple-route models to include explicit pathways for early semantic processing. Their findings were supported by Arcara et al. (2014) reporting increased processing cost for head-final compared to head-initial Italian compounds, visible in an enhanced LAN component.

In sum, the literature points to a certain amount of routine decomposition, or put differently, to direct access to the constituents of complex words during word recognition. This is strikingly visible in the compound benefit, i.e., a processing advantage of compounds relative to simple words matched for length and frequency, if the compounds contain highly accessible constituents. This has been shown for lexical constituent properties like frequency (Bronk et al., 2013). However, it is still unclear if semantic constituent properties are also accessed during compound recognition. The existing literature on semantic decomposition deals with semantic properties that only apply in the context of the compound word, like headedness (in languages like Italian or Basque) or semantic transparency. While this line of research has added important insight to our understanding of compound processing, the manipulations in the stimulus material always concerned semantic contributions of constituents to the full-form meaning. This type of semantic property is not a semantic property of the constituent noun *per se*, and is unlikely to be part of its lexical entry. Therefore, if we aim to answer the question whether semantic constituent properties play a role during compound recognition (in parallel to the role played by lexical constituent properties like frequency), we need to manipulate an intrinsic semantic constituent property that is independent of the compound context and allows us to measure semantic constituent access directly.

This is where the animacy effects outlined above can add important insight: Animacy is an intrinsic semantic property of the constituents, and can be manipulated independently for constituents and the full form. To illustrate, the compounds *Wolfshund* (“wolfhound”) and *Schlittenhund* (“sled dog”) both refer to animates, as their lexical heads refer to animates. These words should be expected to have a processing advantage over compounds referring to inanimates, like *Pferdedecke* (“horse rug”) and *Tischdecke* (“table cloth”). However, for *Wolfshund*, both the modifier and the lexical head are animate, whereas for *Schlittenhund*, the modifier is inanimate. This leads us to formulate the following general research hypotheses:

1. If semantic constituent properties play a role in compound recognition, we would expect a processing advantage for *Wolfshund* (full-form animate with animate modifier) over *Schlittenhund* (full-form animate with inanimate modifier). In a similar vein, we would expect a processing advantage of *Pferdedecke* (full-form inanimate with animate modifier) over *Tischdecke* (full-form inanimate with inanimate modifier).
2. However, if lexical, but not semantic constituent properties play a role in compound recognition, we would expect no processing advantage for *Wolfshund* over *Schlittenhund*, since both full forms refer to animates. Neither would we expect a processing advantage for *Pferdedecke* over *Tischdecke*, since both full forms refer to inanimates. Instead, we would expect to see a clear processing advantage of full-form animates (*Wolfshund* and *Schlittenhund*) over full-form inanimates (*Pferdecke* and *Tischdecke*), without any influence of modifier animacy.

### 2.3. Research questions and hypotheses

The current study is designed to answer the research question whether semantic constituent properties are accessed during compound recognition. To this end, we monitor single word recognition of simple words and compounds in a lexical decision task using EEG measurements.^9^ Based on the literature, we assume that the most reliable indicator of the processing cost associated with lexical accessibility (in general and to compound constituents) is the N400 amplitude [see Kutas and Federmeier (2000, 2011) for lexical accessibility in general and Vergara-Mart́ınez et al. (2009) and Holle et al. (2010) for constituent accessibility in particular]:

- For single words, we assume a straightforward link between animacy and N400 amplitude - animate simple words should elicit reduced N400 amplitudes than inanimate simple words. This comparison serves as our control to replicate basic findings from the literature and ensure that our measurements are sensitive enough to spot processing differences between existing words brought on by semantic factors.
- For compounds, we assume a link between lexical accessibility and N400 amplitude.
  - If lexical constituent properties are *not* accessed during compound recognition, we expect the N400 amplitude to reflect full-form animacy, which is identical with the animacy of the head.
  - If lexical constituent properties are accessed during compound recognition, we expect the N400 amplitude to reflect both the animacy of the lexical head (identical with full-form animacy) and the modifier.

## 3. Language materials

Language materials consisted of one set of simple words (the control conditions) and another set of compounds (the critical conditions). All words were German nouns. Words were interspersed with non-words resembling simple words and compounds. Non-words followed the rules of German phonotactics and orthography, but at the same time were not designed to be particularly difficult to spot or to contain existing words as their constituents. Examples of simple pseudowords include *Schapf* or *Lofer*; examples of compound pseudowords include *Bopfhalz* or *Pluserfeun* (none of these words have a meaning in German, and neither do the pseudo-constituents *Bopf, Halz, Pluser*, and *Feun*). The simple word set had 40 simple words per condition (80 in total) interspersed with 80 simple pseudowords. The compound word set had 40 compounds per condition (160 in total) interspersed with 160 compound pseudowords.

Simple words came in two conditions, inanimate or animate. Animates referred to animals, but not to humans or professions. Inanimates referred to concrete objects, never to abstract concepts. Compounds came in four conditions, named for the animacy of the modifier and the animacy of the head (in this order): inanimate-inanimate, animate-inanimate, inanimate-animate, animate-animate. Full-form animates (conditions inanimate-animate and animate-animate) referred to animals, but not to humans or professions. Full-form inanimates (conditions animate-inanimate and animate-animate) referred to concrete objects, never to abstract concepts. Likewise, animate constituents always referred to animals. Inanimate constituents referred to concrete objects.^10^

Results for simple words were not meant to be compared directly to results for compound words. For this reason, the matching described below was performed for both stimulus sets separately.

### 3.1. Length and frequency matching

Frequencies were accessed from the dlexdb corpus described in Heister et al. (2011) (access: April 2022).^11^

#### 3.1.1. Simple words

Simple words and non-words were matched for length in characters [words = 6.60, non-words = 6.65, *t*_(79)_ = 0.52, *p* > 0.6]. Animate and inanimate words were matched for length in characters [animate = 6.65, s.d. = 1.59, inanimate = 6.55, s.d. = 1.48, *t*_(39)_ = 0.22, *p* > 0.8], and lemma frequency [animate = 250.28, s.d. = 238.06, inanimate = 245.20, s.d. = 183.20, *t* = −0.62, *p* > 0.5].

#### 3.1.2. Compounds

Full forms of compound words and non-words were matched for length in characters [words = 10.19, s.d. = 1.66, non-words = 9.81, s.d. = 1.38, *t*_(159)_ = 2.50, *p* > 0.01].^12^

Matching for compound words was performed using 2 × 2 ANOVAS with the factors modifier and head animacy. Compound words were matched for full-form length in characters (animate-animate = 10.05, s.d. = 1.55, animate-inanimate = 10.50, s.d. = 1.26, inanimate-animate = 10.20, s.d. = 1.94, inanimate-inanimate =10.03, s.d. = 1.82, no statistically significant differences). They were also matched for full-form lemma frequency [animate-animate = 26.15, s.d. = 38.08, animate.inanimate = 24.27, s.d. = 38.12, inanimate-animate = 30.82, s.d. = 43.84, inanimate-inanimate = 33.23, s.d. = 41.51; modifier *F*_(1,56)_ = 1.09, *p* > 0.2; head *F*_(1,56)_ = 2.64, *p* > 0.1; modifier:head *F*_(1,56)_ = 0.17, *p* > 0.6].

In addition, compound words were matched for lengths and lemma frequencies of heads and modifiers. For modifiers, there were no significant effects and interactions of modifier and head on length [mean values: animate-animate 4.75, s.d. = 1.08, animate-inanimate = 4.90, s.d. = 1.08, inanimate-animate = 5.05, s.d. = 1.13, inanimate-inanimate = 5.00, s.d. =1.06; modifier *F*_(1,56)_ = 1.50, *p* > 0.2; head *F*_(1,56)_ = 0.17, *p* > 0.6; modifier:head *F*_(1,56)_ = 0.31, *p* > 0.5] and lemma frequency [mean values: animate-animate = 3905.07, s.d. = 13684.31, animate-inanimate = 3078.18 s.d. = 3408.76, inanimate-animate = 3282.30, s.d. = 2740.30, inanimate-inanimate = 3240.22, s.d. = 3467.06; modifier *F*_(1,56)_ = 2.30, *p* > 0.1; head *F*_(1,56)_ = 2.35, *p* > 0.1; modifier:head *F*_(1,56)_ = 2.58, *p* > 0.1].

For heads, there were no significant main effects or interactions of modifier and head on length [mean values: animate-animate = 4.75, s.d. = 1.08, animate-inanimate = 4.92, s.d. = 0.97, inanimate-animate = 4.95, s.d. = 1.58, inanimate-inanimate = 5.03, s.d. = 1.13; modifier *F*_(1,56)_ = 0.29, *p* > 0.5; head *F*_(1,56)_ = 0.85, *p* > 0.3; modifier:head *F*_(1,56)_ = 0.01, *p* > 0.9] and lemma frequency [mean values: animate-animate = 1741.03, s.d. = 2217.59, animate-inanimate = 1748.03, s.d. = 2205.65, inanimate-animate = 2158.55, s.d. = 3219.34, inanimate-inanimate = 1945.70, s.d. = 1679.16; modifier *F*_(1,56)_ = 1.86, *p* > 0.1; head *F*_(1,56)_ = 1.97, *p* > 0.1; modifier:head *F*_(1,56)_ = 0.12, *p* > 0.7].

In addition, compounds were matched for Levenshtein neighborhood sizes, extracted from the dlexdb corpus (see Laszlo and Federmeier, 2011 for the link between neighborhood sizes and N400 amplitude). We collected the numbers of higher-frequency neighbors (HF neighbors) and the total number of neighbors (all neighbors) for constituents and full-forms. For full-forms of compounds, there were only 27 items of the 160 which had 1 higher-frequency neighbor. The remaining had none. We therefore refrained from an analysis of full-form neighborhood sizes.

For modifiers, there were no significant effects or interactions of head animacy and modifier animacy for the mean number of all neighbors. For the mean number of more highly frequent neighbors, there was a main effect of modifier animacy [*F*_(1,156)_ = 5.7, *p* < 0.5]. For noun-noun compounds with animate modifiers, the mean number of HF neighbors was 2.4. For noun-noun compounds with inanimate modifiers, the mean number of HF neighbors was 1.4 (numbers only take into account the modifiers that did have HF neighbors). We are confident that this small numerical difference does not put us at risk of a systematic confound. (Inanimate-inanimate: mean HF neighbors = 1.6, mean all neighbors 32.7, 24 items had more highly frequent neighbors; animate-inanimate: mean HF neighbors = 2.0, mean all neighbors = 33.0, 28 items had more highly frequent neighbors; inanimate-animate: mean HF neighbors = 1.2, mean all neighbors = 2.8, 29 items had more highly frequent neighbors; animate-animate: mean HF neighbors = 2.8, mean all neighbors = 36.7, 20 items had more highly frequent neighbors).

For heads, there were no significant effects or interactions of head animacy and modifier animacy. This held for both the mean numbers of more highly frequent neighbors and the mean numbers of all neighbors. (Inanimate-inanimate: mean HF neighbors = 2.3, mean all neighbors = 36.1, 28 items had HF neighbors; animate-inanimate: mean HF neighbors = 3.1, mean all neighbors = 36.4, 31 items had HF neighbors; inanimate-animate: mean HF neighbors = 3.3, mean all neighbors = 34.8, 34 items had HF neighbors; animate-animate: mean HF neighbors = 2.9, mean all neighbors = 36.5, 34 items had HF neighbors).

### 3.2. Familiarity

In a prestudy, the stimuli were rated for familiarity by 10 monolingually raised native German speakers (seven male, three female, mean age = 26.5 years, s.d. = 5.64 years, max = 34 years, min = 19 years). Ratings were elicited on a four-paint scale containing the ratings *kenne ich* (‘I know [this word]'), *verstehe ich/habe ich schon einmal gehört* (‘I understand [this word]/I have heard [this word] before') and *kenne ich nicht* (‘I do not know [this word]').

#### 3.2.1. Simple words

For words, the mean number of ‘I know' ratings was 9.7 (minimum number of ‘I know' ratings for any single word was 8); mean number of ‘I understand' ratings was 0.1 (max. was 1); mean number of ‘I don't know' ratings was 0.2 (maximum number for a single word was 2). For non-words, the mean number of ‘I don't know' ratings was 9.2 (minimum number for a single non-word was 6), mean number of ‘I understand' ratings was 0.6 (maximum 4), mean number of ‘I know' ratings was a 0.3 (maximum 2).

#### 3.2.2. Compounds

For words, the mean number of “I know” ratings was = 9.5 (with the minimum of a single word being 4); mean number of “I understand” ratings was 0.3 (max = 4); mean number of “I don't know” ratings was = 0.15 (max = 3). For non-words, the mean number of “I know” ratings was 0.1 (max = 2), the mean number of “I understand” ratings was 0.3 (max = 3), and the mean number of “I don't know” ratings was 9.6 (min = 6). This indicates that simple and compound words were familiar to participants, and that simple and compound non-words clearly recognizable as non-words and did not resemble existing words.

### 3.3. Semantic transparency

To ensure that our compound nouns could truly be considered semantically transparent, we conducted a transparency rating study. Compounds were interspersed with 40 filler items, namely, compounds that we expected to be semantically intransparent, to provide participants with a contrast between transparent and opaque compounds (remember that our stimulus set did not contain semantically opaque compounds). Intransparent compound nouns included *Muskelkater* (lit. ‘muscle cat', a muscle ache after exercise), *Schlafmütze* (lit. ‘sleep hat', i.e., a sleepy head) or *Milchstraße* (lit. ‘milk street', i.e., the Milky Way). Transparency ratings were given by 10 monolingually raised native German speakers (mean age 25.4 years, s.d. = 3.9 years, min = 18, max = 30; five male, five female). Ratings were given on a three-point scale with the points ‘transparent', ‘unsure', and ‘not transparent'. For transparent compounds, the mean across conditions for ‘transparent' ratings was 8.7 (out of 10 raters). In comparison, for opaque compounds, the mean rating for ‘transparent' was 1 and ‘not transparent' was 8.05. Thus, all our compounds are clearly rated differently from truly opaque compounds.

## 4. Experiment

### 4.1. Methods

#### 4.1.1. Participants

Forty participants were recruited via SONA systems database at the University of Konstanz. All of them were monolingually raised native speakers of German. They had normal or corrected to normal vision and reported no history of psychological or neurological illness. All participants were right-handed as assessed by the Edinburgh Handedness Inventory (Oldfield, 1971). The measurement was conducted in accordance with COVID-19 health safety regulations. All parties involved in the experiment were wearing medical masks and had negative antigen test results not older than 24 h before the arrival to the laboratory. Participants signed an informed consent form and received 25 € compensation for their time. The research was approved by the Research Ethics Committee of the University of Konstanz under the ethics approval number 05/2021. During data analysis, one participant was excluded due to poor data quality. The remaining 39 participants had ages ranging from 18 to 31 years (mean = 23.2 years, s.d. = 2.8 years). 18 participants were male, 22 participants were female.

#### 4.1.2. Procedure

The EEG was recorded with BrainVision Recorder (version 1.24.0001, Brain Products GmbH), with 64 EEG actiCAP slim electrodes, attached to an elastic cap with actiCAP SNAP holders and connected to BrainAmp DC amplifiers. The electrode arrangement was based on the equidistant M43-V1 layout as provided by Easycap GmbH. Horizontal and vertical eye movements were registered by four EOG Ag/AgCl sintered passive ring electrodes, connected to BrainAmp ExG bipolar amplifier. Data were recorded in the frequency range 0.016–250 Hz. Impedance values below 20 kΩ were accepted. The signal was digitized with a sampling rate of 500 Hz. Participants were comfortably seated in a sound-attenuated room in front of a monitor at approximately two meters. They were asked to avoid excessive eye and body movements during the EEG recording. They were instructed to press the right button if a word was presented on the screen, and the left one if there was a non-word. During the measurement, no feedback regarding the correctness of their response was given. The Presentation software by Neurobehavioral Systems Inc. (version 20.2) was used for delivering stimuli and trigger codes. Depending on the button press reaction time, the presentation of stimuli took approximately 18–20 min. It was divided into three runs with short breaks between them.

#### 4.1.3. Data preprocessing and analysis

Data were processed using the Brain Vision Analyzer 2 software (Brain Products, Gilching). Raw data were inspected visually, and time windows including strong visible artifacts, as well as breaks, were manually removed. Next, we performed an ICA blink correction using the slope algorithm, followed by filtering (low cutoff 0.5 Hz, high cutoff 40 Hz, 50 Hz notch filter) and topographic interpolation via triangulation for channels showing long stretches of noisy data. After interpolation, all electrodes were re-references to average reference. An Automatic Raw Data Inspection was performed for the re-referenced data (maximal allowed voltage step: 50 μ/ms; maximal allowed difference: 100 μV/200 ms; minimal/maximal allowed amplitudes 200 μV/−200 μV; lowest allowed activity: 0.5 μV/100 ms). Data were segmented starting at 100 ms before stimulus onset and ending at 800 ms after stimulus onset. A baseline correction was performed for 100 ms before stimulus onset. Averages were calculated per participant for all conditions. Participants with less than 35 trials in one of the six experimental conditions were excluded from the data analysis, leading to the exclusion of one participant.

We exported mean amplitudes per condition for each participant from the time window from 350 to 450 ms. This time window was chosen based on the literature, as well as the fact that the peak of the N400 component was close to or around 400 ms, supporting our assumption that the classical N400 is a relevant component to monitor lexical access.

For analysis a subset of 25 electrodes was selected. Electrode position was coded by assigning electrodes to five medial-lateral as well as five anterior-posterior positions. Medial-lateral positions were: lateral-left (front to back: F7, FC5, C5, P5, PO7), medial-left (front-to-back: F3, FC3, C3, CP3, PO3), midline (front-to-back: Fz, FCz, Cz, CPz, Pz), medial-right (front-to-back: F4, FC4, C4, CP4, PO4), and lateral-right (front-to-back: F8, FC6, C6, P6, PO8). Anterior-posterior positions were: anterior (left to right: Fz, F3, Fz, F4, F8), medial-anterior (left to right: CF5, FC3, FCz, FC4, FC6); medial (left to right: C5, C3, Cz, C4, C6), posterior-medial (P5, CP3, CPz, CP4, P6), posterior (left to right: PO7, PO3, Pz, PO4, PO8).

We performed a repeated measures ANOVA of the mean voltages in the selected electrode sites. Only voltages elicited by words were analyzed. For simple words, we performed a repeated-measures ANOVA monitoring the main effects and interactions of within-subjects factors animacy, anterior-posterior position, and medial-lateral position. For compounds, we performed a repeated-measures ANOVA monitoring the main effects and interactions of within-subjects factors modifier animacy, head animacy, anterior-posterior position, and medial-lateral position. Statistical analyses were performed in a hierarchical fashion, i.e., only statistically significant interactions were pursued, unless specifically mentioned otherwise. Interactions were resolved if they included at least one of the experimental factors (animacy for simple words, modifier or head for compounds). A Huyhn-Feldt correction was performed when the degree of freedom in the numerator was higher than 1. Original degrees of freedom and corrected probability levels are reported. Analyses were performed in R (R Development Core Team, 2019) using the ezANOVA function of the ez package (Lawrence, 2011).

### 4.2. Results

For the sake of readability, we only report the highest interactions involving the experimental factors, followed by the resolution of these interactions.

#### 4.2.1. Simple words

Descriptively speaking, waveforms were more negative-going for the inanimate than for the animate condition. This was most visible over central and posterior sites. The negativity for inanimate conditions was clearly visible around 400 ms, and persisted up until around 600 ms; the later negativity was more pronounced in posterior than central sites.

In the time window from 350 to 450 ms, there was an interaction of animacy and medial-lateral [*F*_(4, 152)_ = 1.31, *p* < 0.01, HF ε = 0.59; animacy significant in medial-lateral regions medial-left (*p* < 0.001), midline (*p* < 0.001), medial-right (*p* < 0.05)].

**Figure 1:**
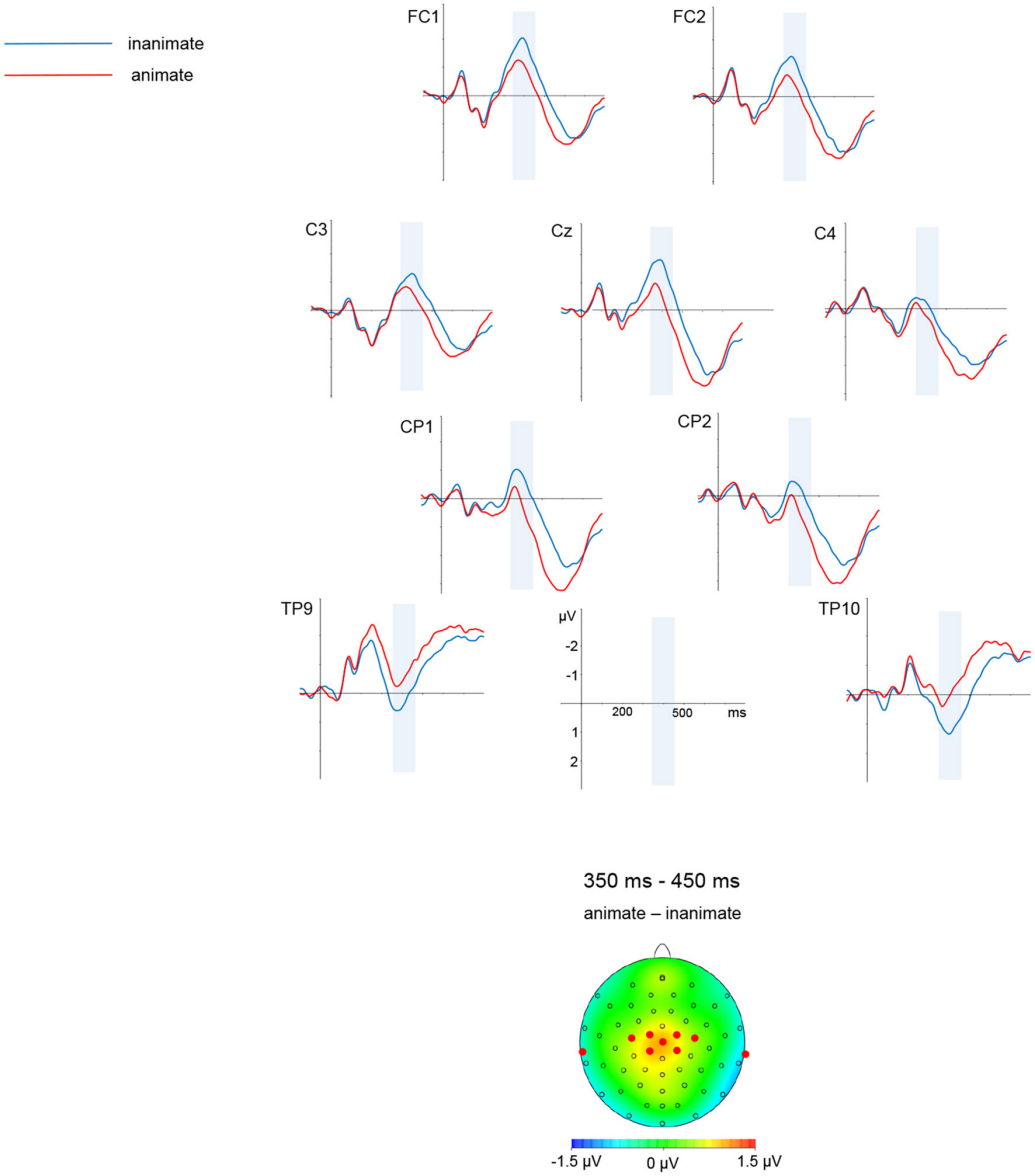
Simple nouns. Grand average ERPs for selected electrode sites and a difference map are shown. A mean voltage difference map (animate minus inanimate) for the marked time window from 350 to 450 ms is given on the left side. The electrodes selected for illustration are marked in the maps.

**Figure 2:**
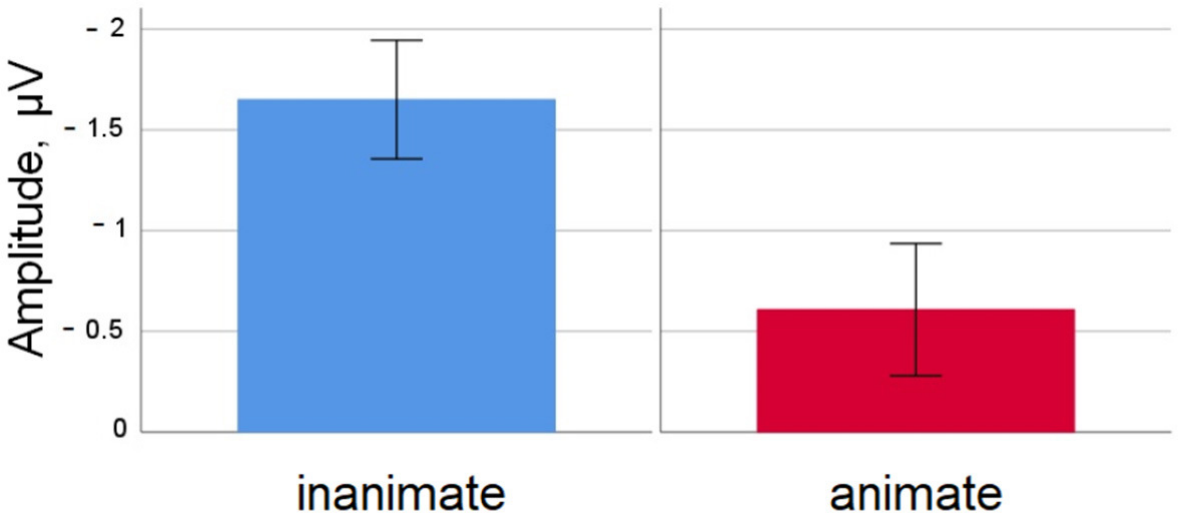
Simple nouns. Mean amplitude difference across participants for the inanimate and animate condition at electrode Cz in the 350–450 ms time window. Error bars depict standard errors of the mean.

A graphic depiction of grand averages for selected electrode sites and voltage difference maps is given in Figures 1, 2 shows mean voltage amplitudes for both conditions at electrode site Cz.

#### 4.2.2. Compounds

Descriptively speaking, waveforms for the inanimate-inanimate condition were more negative-going than for the animate-animate condition. This was most visible over central and posterior sites; the negativity was clearly visible around 400 ms and persisted until about 600 ms. While the general pattern was similar to findings for the simple nouns, the amplitude differences for compounds were rather smaller. The waveforms for the mixed conditions animate-inanimate and inanimate-animate ran mostly together, between the inanimate-inanimate and animate-animate waveforms. The general pattern was that the number of animate constituents was reflected in the amplitude, with waveforms going more positive for each animate constituent. There was no visible influence of the type of constituent that was animate (no stronger influence of head and thereby full-form animacy compared to modifier animacy).

In the time window from 350 to 450 ms, there was a significant main effect of head [*F*_(1,38)_ = 11.20, *p* < 0.01], and an interaction of modifier and anterior-posterior position [*F*_(4, 152)_ = 3.85, *p* < 0.05, ε = 0.39; modifier significant in anterior-posterior regions medial (*p* < 0.01), posterior-medial (*p* < 0.01), and posterior (*p* < 0.05)].^13^

**Figure 3:**
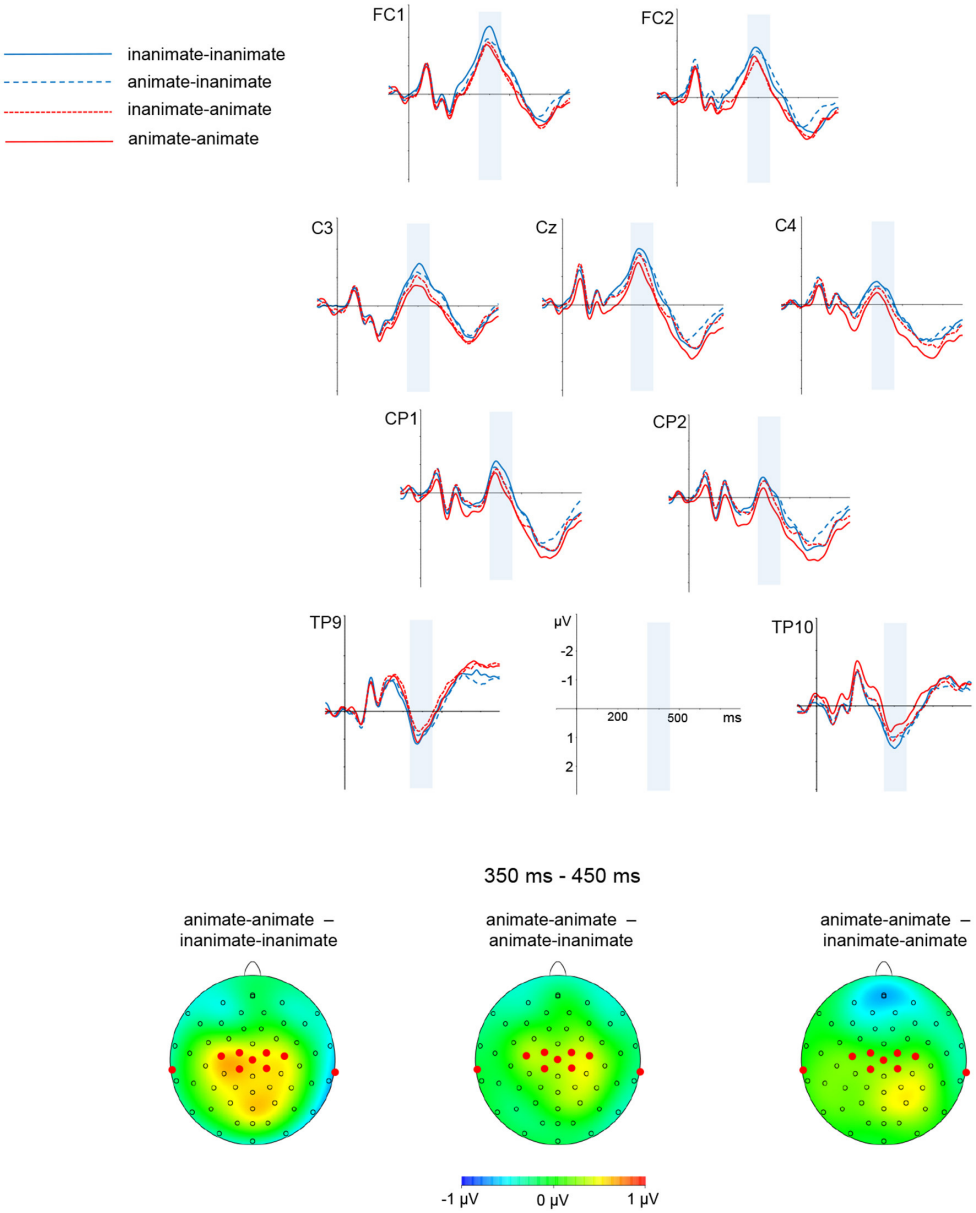
Noun-noun compounds. Grand average ERPs for selected electrode sites and difference maps are shown. Mean voltage difference map (animate-animate minus each of the other conditions) for the marked time window from 350 to 450 ms is given on the left side. The electrodes selected for illustration are marked in the maps.

**Figure 4:**
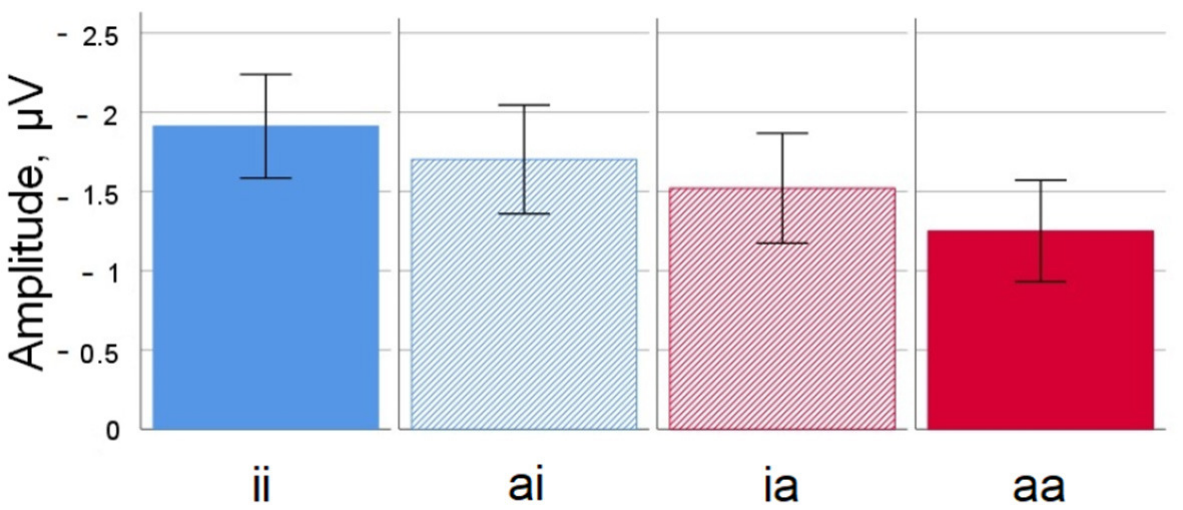
Noun-noun compounds. Mean amplitude difference across participants for all four conditions at electrode Cz in the 350–450 ms time window. Error bars depict standard errors of the mean. ii, inanimate-inanimate; ai, animate-inanimate; ia, inanimate-animate; aa, animate-animate.

A graphic depiction of grand averages for selected electrode sites and voltage difference maps is given in Figures 3, 4 shows mean voltage amplitudes for all four conditions at electrode site Cz.

## 5. Discussion and conclusion

For simple words, the N400 had a larger amplitude for inanimates than for animates. This fits findings from the literature indicating that lexical access is more costly for inanimate compared to animate nouns that are matched on lower-level factors like frequency and length (Janyan and Andonova, 2011; Nairne et al., 2013; Bonin et al., 2014, 2015, 2019; VanArsdall et al., 2015, 2017; Bugaiska et al., 2019), and that this reduced processing cost for animates surfaces as a reduced N400 amplitude (Proverbio et al., 2007). The difference in N400 amplitudes already becomes visible in a simple lexical decision task, without additional tasks like semantic categorization (as in Proverbio et al., 2007) needed. This shows that the influence of animacy on lexical accessibility is robust even in routine single-word processing, and that the N400 amplitude is an informative measure to tap into this.

For compounds, both head animacy (which corresponds to full-form animacy) and modifier animacy influence the N400 amplitude. The amplitude differences are smaller than for simple words, but reach statistical significance. Generally speaking, the N400 amplitude is least negative-going for animate-animate compounds, and most negative-going for inanimate-inanimate compounds. Compounds with one animate and one inanimate constituent show an N400 that tends to run between these two extremes. Neither a descriptive overview nor the statistical analysis suggest an interaction between modifier and head animacy. At this point, the facilitating effect of constituent animacy seems to be additive - the higher the proportion of animate constituents, the less negative-going the N400 amplitude will be. Descriptively, the distribution both head and modifier effects fits with the usual N400 topography. However, the interaction with topographical factors was only significant for modifier animacy.

Our findings strongly support the idea that constituent properties of (transparent) compounds are routinely accessed during compound recognition [see, a.o., Pollatsek et al. (2000), Juhasz et al. (2003), Andrews et al. (2004), Fiorentino and Poeppel (2007), Kuperman et al. (2009), Wang et al. (2010), and in particular Bronk et al. (2013)].

Unlike some earlier studies (Duñabeitia et al., 2007; Vergara-Mart́ınez et al., 2009; Holle et al., 2010), our current findings do not support a privileged role for the second constituent, which in German always coincides with the lexical head. While the influence of head animacy was more widespread than the role of modifier animacy, both were present and statistically significant in the N400 time window. The absence of an interaction between modifier and head animacy further supports the idea that during the investigated time window, N400 amplitude simply reflects the added accessibility, which is mainly influenced by the accessibility of the constituents, without visible overriding influences of head/full-form animacy. Discrepancies to these earlier studies may stem from the differences in possible headedness in Basque and Spanish [in the case of Duñabeitia et al. (2007) and Vergara-Mart́ınez et al. (2009)], or due to differences in stimulus design (our own stimuli did not contain opaque compounds, while there is no mention for control of constituent properties across conditions in Holle et al., 2010).

Most importantly in the context of our research question, our findings strongly support the idea that semantic properties of both heads and modifiers influence lexical accessibility. This is in contrast to some earlier studies arguing against semantic access to compound constituents (Duñabeitia et al., 2007) during decomposition, but fits the wider literature describing some amount of semantic (de)composition for compounds [see Sandra (1990), Zwitserlood (1994), and Koester et al. (2007, 2009) for transparent compounds, Smolka et al. (2014) and Smolka and Eulitz (2018) for both transparent and opaque compound verbs, and Ji et al. (2011) for a nuanced discussion of semantic composition in transparent and opaque noun-noun compound recognition]. Unlike these early studies, our findings provide evidence for the direct influence of intrinsic semantic constituent properties via our manipulation of constituent animacy, allowing a direct comparison to manipulations of lexical constituent properties (like e.g., frequency in Bronk et al., 2013). Our studies thus support the earlier findings on lexical decomposition, but circumvent some of the problems stemming from the indirect approach to semantics (via, e.g., semantic transparency; see MacGregor and Shtyrov, 2013 and also Koester et al., 2007 for insightful discussions on the difficulties of interpreting absent priming effects for opaque compounds, since they could either reflect the fact that semantic constituent priming does not happen in opaque compounds because they are accessed differently, or that it does not happen because prime and target are not semantically related by virtue of the compound being opaque.).

Importantly, our results were found in a context that did not particularly encourage semantic or morphological decomposition, and in comparison of words to “easy” to spot pseudowords that respected the rules of German orthography and phonotactics, but did not need careful reading and did not combine existing with non-existing constituents. We therefore interpret our findings as showing that access to semantic constituent properties is an automatic and routine process during the recognition of semantically transparent compounds (in contrast, to e.g., Stites et al., 2016 in the more recent literature).

With respect to models of word recognition, our findings would fit into full-parsing models (e.g., Taft and Forster, 1975; Libben et al., 1999 and follow-up models), but also into dual/multiple route models (Caramazza et al., 1988; Baayen and Schreuder, 1999; Kuperman et al., 2009), since they allow constituent access for transparent compounds. A prerequiste is that the models allow for early access to semantic constituent properties in addition to lexical ones, supporting the demands in Marelli and Luzzatti (2012) for formulating explicit extensions for early semantic processing pathways in the context of existing multiple-route models.

Future studies should address issues of timing differences between full-form and constituent access to elucidate if one precedes the other. It would also be informative to monitor semantic composition in semantically opaque compounds using the N400 amplitude as a direct measure of the ease of lexical access, and constituent animacy manipulations to influence the relative difficulty of said access. However, this would also imply a careful control of potential confounding factors like the semantic relation between modifier and head across conditions, and elegant solutions to disentangle absent effects from multiple overlaying effects for semantically opaque compounds (see Ji et al., 2011 for a detailed discussion of this issue).

For future studies on the effects of headedness and constituent position (in continuation of Duñabeitia et al., 2007; El Yagoubi et al., 2008; Vergara-Mart́ınez et al., 2009; Marelli and Luzzatti, 2012; Arcara et al., 2014), animacy manipulations may provide a useful tool to monitor semantic access and its interactions with headedness in languages like Basque or Italian, allowing a more direct approach than the one taken in previous studies via manipulations of semantic transparency.

In general, our findings show the strong influence that constituent animacy has on lexical accessibility in compound recognition, visible in related neurolinguistic measures. Irrespective of specific research questions, this shows that along with lexical constituent properties like length and frequency, semantic constituent properties like animacy need to be carefully controlled and/or balanced in studies investigating compound processing to avoid losing significant effects, or even end up with spurious effects in severely unbalanced stimulus sets. In a similar vein, in sentence processing research, it is advisable to control for morphological complexity of words in comparable positions across conditions, taking into account full-form and constituent properties alike, to avoid contamination from complex single-word recognition effects.

## Data availability statement

The raw data supporting the conclusions of this article will be made available by the authors upon request, without undue reservation.

## Ethics statement

The studies involving humans were approved by Ethikkommission der Universität Konstanz (Ethics Committee of the University of Konstanz). The studies were conducted in accordance with the local legislation and institutional requirements. The participants provided their written informed consent to participate in this study. No potentially identifiable images or data are presented in this study.

## Author contributions

AC was responsible for experiment conception and design, running data preprocessing and statistical analysis, as well as for overseeing data acquisition. MK was responsible for discussing choices in data preprocessing and analysis and for overseeing data acquisition. CE was responsible for experiment conception, discussing choices in data preprocessing and analysis, as well as for providing laboratory infrastructure. All authors contributed to writing the manuscript.

## Footnotes

^1^From Latin anima, “the soul”.

^2^For the sake of readability, we will use “animate/inanimate” to also refer to “words with animate/inanimate referents”. In case a clear distinction between referents and words referring to them is necessary, we will be more specific.

^3^Please refer to the Supplementary material for background information on the ERP literature and on the P300 and N400, the two ERP components that are most relevant in the context of the current study.

^4^For the sake of readability, we do not give an in-depth overview of the role of animacy in sentence processing at this point. We will discuss the implications of our findings for sentence processing research in the discussion.

^5^While some studies deal with changes of perceived animacy/agency, e.g., Nieuwland and Van Berkum (2006), these studies are specifically designed to override a noun's intrinsic animacy via an elaborate discourse context.

^6^Compounding is not limited to two nouns; examples for longer German compounds range from everyday words like Rechts.schutz.versicherungs.makler, law.protection.insurance.broker, ‘legal defense insurance broker’; to unusually long examples like ‘Weihnachts.baum.schmuck.ausstattungs.spezial.geschäft’, literally ‘christmas.tree.decoration.equipment.special.shop’, to be found on a shopfront in the Swiss city of Basel; see https://de.wikipedia.org/wiki/Johann_Wanner_Unternehmer for illustration.

^7^Our work is rooted in the combinatorial tradition of research on the mental lexicon. Please refer to the Supplementary material for a brief discussion of links to learning-based models of lexical access.

^8^For the sake of readability and brevity, we limit this literature overview mainly to work on compound recognition, omitting the vast literature on the processing of derived complex words. An overview of the general parallels and differences is given in Leminen et al. (2019).

^9^First tentative evidence in favor of semantic constituent access comes from behavioral studies in the context of two qualification theses at the University of Konstanz (Strinzel, 2014; Gozebina, 2017). These studies used a visual lexical decision task, following the general approach that was used in Bronk et al. (2013) tomonitor lexical decomposition. The general direction of findings was that both modifier and head animacy influenced reaction times, supporting the idea that semantic constituent properties are accessed during recognition. However, due to issues with stimulus control and the small scale of the studies, the findings are not conclusive. The goal in our current study is to monitor the extent to which semantic constituent properties have an influence on lexical accessibility during compound recognition, using a carefully controlled stimulus set and EEG measurements.

^10^We avoided words with abstract referents, since contrasts between abstract- and concrete-referring words are known to influence N400 amplitude (West and Holcomb, 2000; Huang and Federmeier, 2015).

^11^We chose to not control for family size, i.e., the numbers of nouns that modifiers and heads combined with to form other noun-noun compounds. Certain types of noun-noun compounds are either over- or underrepresented in the current version of the DWDS corpus (to our knowledge the most extensive German corpus for our purposes), making the available data unreliable. Since family size is unlikely to have a strong influence on N400 amplitudes (Kwon et al., 2012), we are confident that this will not be a major concern.

^12^The mean length difference between compound words and non-words was smaller than one character, but still reached statistical significance. Since non-words were only added to the experiment to provide a task in lexical decision, but were not meant to be analyzed or compared to words, we decided to accept this small length difference.

^13^Following the recommendation of an anonymous reviewer, we reanalyzed the data with a reduced participant set, excluding the data from three participant with a high number of interpolated channels (8 or 9 out of 64 channels). While reducing the participant set reduces the statistical power and turned the interactions with the multi-level topographical factors into marginally significant ones, the main effect of modifier now became significant. This shows that the effect ofmodifier animacy cannot be reduced to artifacts introduced by overly interpolated data.
