# Supplementary material for: Wolf-hound vs. sled-dog: neurolinguistic evidence for semantic decomposition in the recognition of German noun-noun compounds
Source: Front Psychol. 2023 Aug 17;14:1173352. doi: 10.3389/fpsyg.2023.1173352 (PMC10470010; doi:10.3389/fpsyg.2023.1173352)
Supplement: Supplementary file 1 [file Data_Sheet_1.pdf]

# Supplementary Material

## SUPPLEMENTARY SECTION

The following supplementary section provides some additional background information that is not included in the main body of the paper in the interest of readability. While we are not giving an extensive and in-depth discussion of these topics here, we hope that the provided references will guide interested readers to the related literature and allow for an easier connection to neighbouring subfields and discussions.

### *EEG and relevant ERP components*

EEG refers to electroencephalography, which is a non-invasive recording of the brain's electrial activity at the scalp. EEG provides multi-dimensional data regarding the polarity, amplitude, and scalp distribution of brain electrical activity elicited by stimuli with millisecond-level resolution. Event-related potentials (ERPs) are EEGs time-locked to the onset of a particular stimulus.

In the context of animacy processing, the two most relevant ERP components are the P300 and the N400. The **P300** is a positive deflection in the EEG waveform, peaking roughly around 300 ms. It is often described as a member of the wider P3 family. Initially described in the 1960ies (Chapman and Bragdon, 1964; Sutton et al., 1965), it has been associated with a high number of different task-related processes, and is known to be related to the processing of oddball stimuli, context updating, attention and possibly decision-making. See Leckey and Federmeier (2020) for a current review of language-related positivities including the P300, and Swaab et al. (2012) for an overview of language-related ERP components in general, including the P3 family and the N400.

The **N400** is a negative deflection in the EEG waveform peaking around 400 ms post stimulus onset, first described by Kutas and Hillyard (1980). In the context of language processing, it has been linked to increased processing cost due to a number of factors, the uniting aspect being increased lexical access cost (Brouwer et al., 2012) or, more neutrally put, "the relative efficiency in processing stimulus or word properties relative to the preceding context" (Alday and Kretzschmar 2019; literature overview in the introduction). Related to this, N400 amplitude is influenced by a host of properties, including a word's frequency, pre-activation via priming or context-based predictions, and it's fit with the sentence and wider discourse context in terms of semantics, plausibility and world knowledge. See Baggio and Hagoort (2011) and Kutas and Federmeier (2011) for thorough reviews and further references, and Hagoort (2013) for its relation to lexical access and semantic unification costs in sentence processing. See also Bayer et al. (2001); Czypionka and Eulitz (2018); Czypionka et al. (2019) for its relation to (non-semantic) lexical case marking effects.

### *Combinatorial and learning-based lexicon models*

Our research question and experiment is rooted in the tradition of combinatorial approaches to the mental lexicon and lexical access. For the sake of brevity, we have omitted the discussion of the alternative tradition of learning-based approaches to the mental lexicon. We recommend Baayen et al. (2011); Harm and Seidenberg (2004) and Milin et al. (2017) for an in-depth overview and comparison of both approaches. Our experiment was designed to pursue a research question from the lexicon-based literature, i.e., to assess whether semantic constituent properties are accessed at all in compound recognition, even in a paradigm that does not actively provoke decomposition. As such, it does not provide a direct link for discussing our

findings within the context of the learning-based approaches to lexical access. However, the learning-based literature concerned with modelling compound processing effects (e.g., Baayen et al. 2011) replicates some of the findings which suggest that constituent frequency influences reaction times in compound recognition. This allows the speculation that constituent property effects like the ones we are reporting in the current paper are possible in principle (even though the notion of morphological ‘constituents’ is not necessary for explaining findings with learning-based models). Once our findings have been replicated for other languages and more research is in place that uses intrinsic semantic constituent properties like animacy, a combination of lexicon-based and learning-based approaches could turn out to be a very fruitful direction for future research.

## REFERENCES

Alday, P. M. and Kretzschmar, F. (2019). Speed-accuracy tradeoffs in brain and behavior: testing the independence of p300 and n400 related processes in behavioral responses to sentence categorization. *Frontiers in human neuroscience* 13, 285

Baayen, R. H., Milin, P., urević, D. F., Hendrix, P., and Marelli, M. (2011). An amorphous model for morphological processing in visual comprehension based on naive discriminative learning. *Psychological Review* 118, 438

Baggio, G. and Hagoort, P. (2011). The balance between memory and unification in semantics: A dynamic account of the n400. *Language and Cognitive Processes* 26, 1338–1367

Bayer, J., Bader, M., and Meng, M. (2001). Morphological underspecification meets oblique case: Syntactic and processing effects in German. *Lingua* 111, 465–514

Brouwer, H., Fitz, H., and Hoeks, J. (2012). Getting real about Semantic Illusions: Rethinking the functional role of the P600 in language comprehension. *Brain Research* 1446, 127–143

Chapman, R. M. and Bragdon, H. R. (1964). Evoked responses to numerical and non-numerical visual stimuli while problem solving. *Nature* 203, 1155–1157

Czypionka, A. and Eulitz, C. (2018). Lexical case marking affects the processing of animacy in simple verbs, but not particle verbs: evidence from event-related potentials. *Glossa: A Journal of General Linguistics* 3. doi:10.5334/gjgl.313

Czypionka, A., Golcher, F., Błaszczak, J., and Eulitz, C. (2019). When verbs have bugs: lexical and syntactic processing costs of split particle verbs in sentence comprehension. *Language, Cognition and Neuroscience* 34, 326–350. doi:https://doi.org/10.1080/23273798.2018.1539756

Hagoort, P. (2013). Muc (memory, unification, control) and beyond. *Frontiers in psychology* 4, 416

Harm, M. W. and Seidenberg, M. S. (2004). Computing the meanings of words in reading: cooperative division of labor between visual and phonological processes. *Psychological Review* 111, 662

Kutas, M. and Federmeier, K. D. (2011). Thirty years and counting: finding meaning in the N400 component of the event-related brain potential (ERP). *Annual Review of Psychology* 62, 621–647

Kutas, M. and Hillyard, S. A. (1980). Reading senseless sentences: Brain potentials reflect semantic incongruity. *Science* 207, 203–205

Leckey, M. and Federmeier, K. D. (2020). The p3b and p600 (s): Positive contributions to language comprehension. *Psychophysiology* 57, e13351

Milin, P., Smolka, E., and Feldman, L. B. (2017). Models of lexical access and morphological processing. In *The Handbook of Psycholinguistics*, eds. E. M. Fernández and H. Smith Cairns (Wiley Online Library). 240–268

Sutton, S., Braren, M., Zubin, J., and John, E. (1965). Evoked-potential correlates of stimulus uncertainty. *Science* 150, 1187–1188

Swaab, T. Y., Ledoux, K., Camblin, C. C., and Boudewyn, M. A. (2012). Language-related ERP components. In *The Oxford Handbook of Event-Related Potential Components*, eds. S. J. Luck and E. S. Kappenman (New York: Oxford University Press). 397–439
